# Ternary Solid Dispersions: A Review of the Preparation, Characterization, Mechanism of Drug Release, and Physical Stability

**DOI:** 10.3390/pharmaceutics15082116

**Published:** 2023-08-10

**Authors:** Arif Budiman, Eli Lailasari, Neng Vera Nurani, Ellen Nathania Yunita, Gracia Anastasya, Rizqa Nurul Aulia, Ira Novianty Lestari, Laila Subra, Diah Lia Aulifa

**Affiliations:** 1 Department of Pharmaceutics and Pharmaceutical Technology, Faculty of Pharmacy, Universitas Padjadjaran, Jl. Raya Bandung-Sumedang Km. 21, Bandung 45363, Indonesia; eli19001@mail.unpad.ac.id (E.L.); neng19001@mail.unpad.ac.id (N.V.N.); ellen20002@mail.unpad.ac.id (E.N.Y.); gracia20001@mail.unpad.ac.id (G.A.); rizqa16001@mail.unpad.ac.id (R.N.A.); 2 Department of Pharmaceutical Analysis and Medicinal Chemistry, Faculty of Pharmacy, Universitas Padjadjaran, Jl. Raya Bandung-Sumedang Km. 21, Bandung 45363, Indonesia; ira19001@mail.unpad.ac.id (I.N.L.); diah.lia@unpad.ac.id (D.L.A.); 3 Faculty of Bioeconomic and Health Sciences, Geomatika University College, Kuala Lumpur 54200, Malaysia; laila@geomatika.edu.my

**Keywords:** ternary solid dispersion, amorphization, poorly water-soluble drugs, amorphous solid dispersions, drug release, physical stability

## Abstract

The prevalence of active pharmaceutical ingredients (APIs) with low water solubility has experienced a significant increase in recent years. These APIs present challenges in formulation, particularly for oral dosage forms, despite their considerable therapeutic potential. Therefore, the improvement of solubility has become a major concern for pharmaceutical enterprises to increase the bioavailability of APIs. A promising formulation approach that can effectively improve the dissolution profile and the bioavailability of poorly water-soluble drugs is the utilization of amorphous systems. Numerous formulation methods have been developed to enhance poorly water-soluble drugs through amorphization systems, including co-amorphous formulations, amorphous solid dispersions (ASDs), and the use of mesoporous silica as a carrier. Furthermore, the successful enhancement of certain drugs with poor aqueous solubility through amorphization has led to their incorporation into various commercially available preparations, such as ASDs, where the crystalline structure of APIs is transformed into an amorphous state within a hydrophilic matrix. A novel approach, known as ternary solid dispersions (TSDs), has emerged to address the solubility and bioavailability challenges associated with amorphous drugs. Meanwhile, the introduction of a third component in the ASD and co-amorphous systems has demonstrated the potential to improve performance in terms of solubility, physical stability, and processability. This comprehensive review discusses the preparation and characterization of poorly water-soluble drugs in ternary solid dispersions and their mechanisms of drug release and physical stability.

## 1. Introduction

The aqueous solubility of a drug holds significant importance in the formulation, particularly in the development of oral dosage forms, since it exerts a substantial influence on bioavailability. After dissolution in gastrointestinal fluid, the molecules are subjected to adsorption from the intestinal sites [1,2]. Over the past two decades, a striking 75% of new chemical entities (NCEs) in pharmaceutical research have exhibited poor solubility in water. Consequently, the formulation of solutions often poses significant challenges due to various issues related to performance [3,4,5,6,7,8]. Conventional methods such as solubilizing agents, complexing agents, and salt formation, have been successfully used to improve the solubility of several poorly water-soluble drugs. These approaches possess limitations in terms of their effectiveness in solubility enhancement, the approved concentration of excipients, and the potential occurrence of different side effects associated with the excipients and co-solvents [9,10,11,12,13]. Therefore, developing a new strategy to improve drug solubility is necessary for the formulation of poorly water-soluble drugs [1].

Amorphization is one of the most promising strategies in drug formulations to increase the solubility, dissolution rate, and bioavailability of poorly water-soluble drugs [14]. Meanwhile, amorphization is the transformation of active pharmaceutical ingredient (API) materials from a crystalline state to an amorphous form. The materials can be synthesized by rapid solidification from the liquid state, the melt method, solvent evaporation, and vapor deposition [15]. Amorphous drugs, owing to their higher Gibbs free energy relative to crystalline counterparts, offer the potential to enhance aqueous solubility and achieve high drug concentrations after dispersion in water [16]. The addition of excipients to inhibit the recrystallization of drugs is necessary for the solid formulation of these drugs since they are thermodynamically unstable and easy to recrystallize after dispersion or during storage [17,18]. 

Some methods have been widely developed to prevent the recrystallization of drugs by the addition of excipients such as amorphous solid dispersions (ASDs) and co-amorphous systems. In the ASD method, the compounds are embedded in the hydrophilic matrix to prevent the crystallization of amorphous drugs [19]. A suitable polymer can stabilize an amorphous drug during storage and maintain high concentrations in the bulk medium through intermolecular interaction [20,21]. Moreover, the presence of a hydrophilic polymer effectively improves the wettability and dissolution rate of the drugs [22]. In the co-amorphous system, the drugs are combined with a small molecule excipient (co-former) that can improve physical stability [23]. The intermolecular interaction between drug and co-former, such as hydrogen bonding [24,25,26] and π–π [27,28], can prevent the conversion of the amorphous drug into the crystalline form [6,29]. The co-amorphous system showed a lower mass of dosage with excellent physical stability compared to the ASD system. The combination of two pharmacologically relevant drugs in this system also has the potential benefits of generating synergic effects and having fewer side effects [4]. However, challenges such as physical stability, wettability, precipitation in dissolution media, and processability have been observed in binary systems. Previous studies reported that approximately 18% of ASD formulations did not demonstrate significant changes in bioavailability during in-vivo analyses involving animals and humans [30]. 

The implementation of ternary solid dispersion (TSD) systems presents a pioneering strategy for augmenting the solubility, wettability, and physical stability of amorphous drugs. By incorporating a third component into the formulation, notable advancements have been observed in reducing the drug’s particle size. This facilitates dissolution and the delivery of the medicament [31]. Therefore, this study aims to summarize and discuss the mechanism of solubility improvement of TSD systems and its impact on pharmaceutical properties. The results are expected to elucidate the molecular pharmaceutic mechanisms of TSDs in the solid state or after being dispersed in water and how these modifications affect solubility, dissolution, and physical stability [32,33].

## 2. Definition of Ternary Solid Dispersion

**Figure 1:**
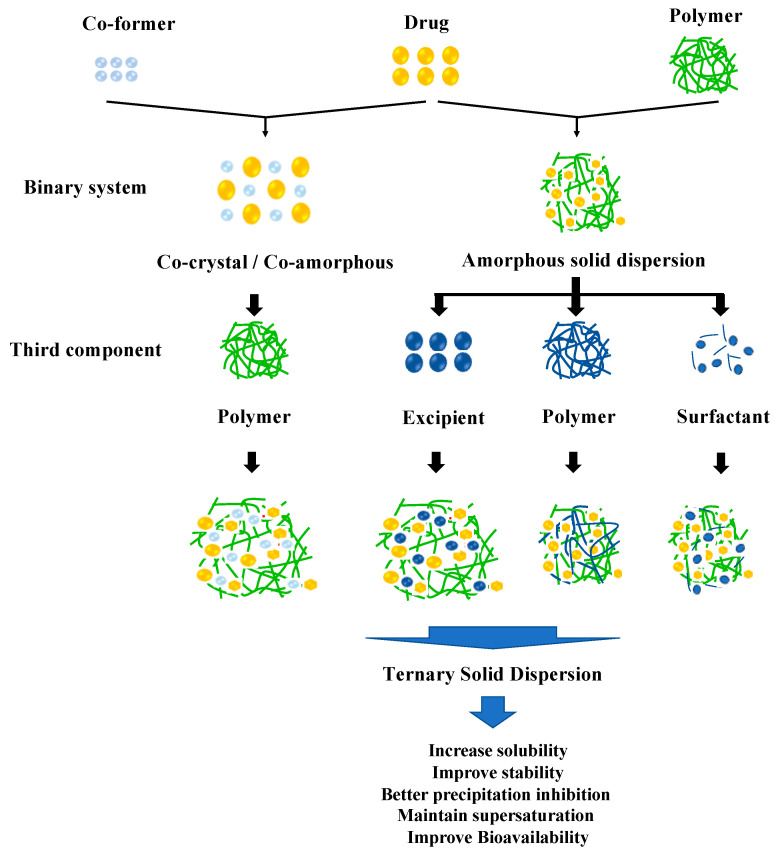
Classification of the TSD system.

A TSD is the solid dispersion system of APIs dispersed into two different excipients at a solid state. Primarily, this method overcomes the problems of pharmaceutical properties such as dissolution profile and physical stability in ASD or cocrystal/co-amorphous systems. The incorporation of a third component, such as a polymer, surfactant, or other compatible excipient, in the drug formulation serves to enhance solubility and promote increased stability. This is achieved through intermolecular interactions between the drug and solubilizer, as well as between the drug and polymer or co-former, resulting in improved solubility and reduced recrystallization [34]. The classification of the TSD system based on the composition can be seen in Figure 1. 

## 3. Components of Ternary Solid Dispersion

### 3.1. ASD + Surfactant

In this system, the TSD system was developed from an ASD system, where the poorly water-soluble drug is embedded in the polymer matrix with the surfactant. The incorporation of a surfactant can facilitate enhanced interaction between the drug and polymer, leading to improved dispersion and superior adsorption [35]. The inclusion of a surfactant enhances the solubility and dissolution of the drug within the medium through the process of coating or the formation of nanomicelles. Previous studies investigating TSDs composed of drug–polymer combinations and surfactants are presented in Table 1. 

Table 1 shows that the presence of a surfactant can help the polymer to achieve high solubility and processability. Anionic surfactants also effectively encapsulate the drug in the matrix of a polymer API system, leading to the improvement of the solubility and dissolution rate of the amorphous drug in the medium [49]. Feng et al. reported that surfactant and hydroxypropyl methylcellulose acetate succinate (HPMC AS) synergistically inhibit the precipitation of amorphous drugs. The surfactant can inhibit thermodynamic and kinetic precipitation, leading to the increasing saturation solubility of amorphous drugs. Furthermore, the adsorption of the surfactant on small particles of the drug results in blockage of the active surface, inhibiting nucleation and crystal growth [50]. 

The presence of a surfactant in the binary system can improve solubility and processability; however, in some cases, it can reduce stability. Baghel et al. reported that the addition of a surfactant to a drug–polymer combination reduced the stability and dissolution by promoting the crystallization of the drug. Another study also reported that the presence of Tween 80 had no effect on the physicochemical properties of the drug and could not improve the dissolution and oral bioavailability significantly. Moreover, the selection of a surfactant for this type in the ternary system is critical. Nonionic surfactants cannot be used in this system because they cannot form ionic bonds with the polymer chains [51,52].

Nevertheless, the addition of the surfactant in ternary solid dispersions can also provide processability benefits. Sometimes the processing of polymers with high glass transition temperatures (*T_g_*) might be difficult in the case of hot-melt extrusion as the polymer will require a high processing temperature, which can degrade other components of the formulation. Surfactants can lower the *T_g_* through the plasticization effect, helping in hot-melt extrusion [53,54].

### 3.2. ASD + Polymer

In this system, the poorly water-soluble drugs were dispersed into two polymers. The addition of more polymers generated different physicochemical properties, improving the performance of amorphous solid dispersion in terms of stability, wettability, and control release. Meanwhile, hydrophilic polymers, such as HPMC and polyvinylpyrrolidone (PVP), were commonly used in TSDs consisting of drug–polymer combinations and polymers, as shown in Table 2. 

Table 2 suggests that the addition of a third component (polymer) to the binary system, such as an amorphous solid dispersion, leads to the improved stability of the formulation. The third component (polymer) acts as a bridge between drug and polymer in ASDs due to the hydrogen-bond interaction. Al-Obaidi et al. (2011) reported that adding a third component (poly[2-hydroxypropyl methacrylate] (PHPMA)) into the solid dispersion of griseofulvin (GF) and PVP can improve the dissolution and wettability properties of griseofulvin via the hydrogen-bond interaction of GF-PHPMA and PVP-PHPMA [65]. Furthermore, Li et. al., reported that the interaction between indomethacin and both Eudragit 100 and PVP K90 synergistically improves the stabilization and dissolution of ASDs. This interaction also inhibits the precipitation and extends the supersaturation of ASDs [51,60]. Adding a third polymer caused high viscosity, contributing to the kinetic retardation of the drug’s molecular mobility. However, the high viscosity of this system revealed low wettability, leading to a slow dissolution rate because the presence of a third polymer suppressed the water contact. Moreover, in some cases, the drugs blended with a polymer show some limitations, such as insolubility at a particular pH [65,66].

### 3.3. ASD + Excipient

Within this particular system, the incorporation of polymers and excipients has been employed to facilitate the preparation of poorly water-soluble drugs. These additives served various purposes, such as functioning as alkalizers, processing aids, or stabilizers, and the excipient was used in TSD systems to overcome the limitations of hydrophilic polymers at a particular pH. Table 3 shows the previous studies that reported TSD systems using drug–polymer combinations and excipients.

Table 3 shows that the addition of excipients into amorphous solid dispersions improved the solubility, stability, and processability of amorphous drugs. Higashi et al. reported that saccharin added to ASDs of probucol and Eudragit stabilized the dispersion system at varied pH levels through the ionic- or hydrogen-bond interaction with the drug and hydrophilic polymer [76]. Dong et al. also stated that the addition of L-arginine as the alkalizer improved the solubility and dissolution of glycyrrhetinic acid in an amorphous state. Moreover, the formation of high-binding-energy ion-pair complexes of glycyrrhetinic acid and L-arginine destroyed the intramolecular H-bond of glycyrrhetinic acid and Kollidon^®^ VA64 as a polymer, increasing the wettability of the drug [67]. However, this system is not possible for the combinations of water-soluble polymers and water-soluble excipients because it is not easy to predict the interaction between the compounds. In addition, 2-hidroksilpropil-β-siklodekstrin (HP-beta-CD) has good capacity and the ability to modify the chemical, physical, and even biological properties of many hydrophobic compounds. However, the large molecular weight and low water solubility of HP-beta-CD leads to the limitation of its application. Thus, an approach to reduce the concentration of effective HP- beta-CD is needed in the formulation of ternary solid dispersions [11,77].

### 3.4. Co-Amorphous/Cocrystal + Excipient

In this system, the poorly water-soluble drugs were prepared with a co-former and polymer. The polymer was added to the TSD system to overcome the limitations of cocrystal or co-amorphous systems, such as solubility, dissolution profile, physical stability, and wettability. Previous studies reported TSD systems using co-amorphous/cocrystal + excipient combinations can be seen in Table 4.

Table 4 shows that the addition of polymer into the cocrystal/co-amorphous system improved the solubility, stability, and wettability of the amorphous drug. Li et al. reported that the molecular mobility of SMZ was reduced by the addition of a polymer, leading to higher stability compared to the binary system. Moreover, the presence of a polymer improved the AUC of SMZ and TMP in their ternary ASDs by 4 and 4.6-fold, respectively [79]. Kosaka et al. also reported that the presence of PVP maintained the supersaturation of IMC despite the drug release of IMC being slower at the beginning of the test compared to the binary system. The different mechanism in the intermolecular interaction and composition was attributed to the improved dissolution profile of IMC in the TSD system [81]. This TSD approach can enhance the stability or dissolution of the amorphous drug via intermolecular interactions. However, in this system, the addition of a polymer in the co-amorphous or cocrystal system can decrease the maximum achievable supersaturation, leading to a slow dissolution rate [82].

A simple comparison of different kinds of ternary solid dispersion can be seen in Table 5

**Figure 2:**
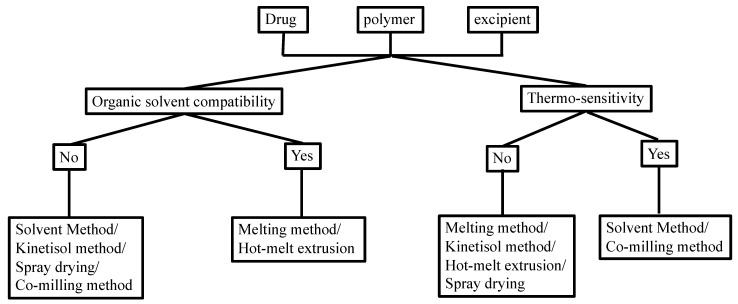
Choosing the preparation method of the TSD system based on the solubility in the organic solvent and thermo-sensitivity. Adapted from data presented originally in Ref. [35]. Copyright 2023 Taylor and Francis.

Manufacturing conditions may significantly affect the physicochemical properties of the TSD formulation. There are some methods that showed satisfactory yields, despite having several challenges with complex TSD preparation. Figure 2 shows the selection of an optimal strategy for TSD preparation, considering the organic solubility and thermal profile of the drugs and each component.

## 4. Characterization of Ternary Solid Dispersion in the Solid State

### 4.1. Fourier Transform Infrared (FTIR)

FTIR is widely used to analyze the intermolecular interactions and reactions between drugs and carriers, specifically in the study of hydrogen bonding between drugs and carriers, with a significant impact on solubility and stability [83]. FTIR can be used to characterize drug–polymer interactions, molecular motions, polymorphs, phase separation, and distinguish between amorphous and crystalline phases [84]. A study on TSDs used this method to detect the interaction between griseofulvin and the carrier. This predicted the improved stability and solubility of dispersion due to the detected hydrogen bonds. Another study employed FTIR to analyze the potential intermolecular interactions between drugs and polymers in TSD formulations of core amorphous systems [85,86].

**Figure 3:**
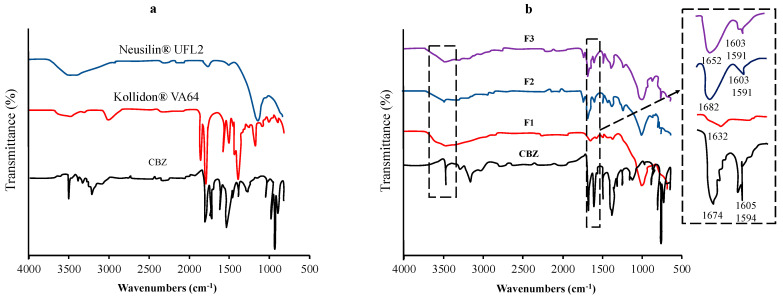
FT−IR spectra of (**a**) each component and (**b**) the samples of the TSD system (F1−20% CBM, 80% Neusilin^®^ UFL2; F2−47.26% CBM, 32.74% Neusilin^®^ UFL2, 20% Kollidon^®^ VA64; F3−44.33% CBM, 39.79% Neusilin^®^ UFL2, 15.87% Kollidon^®^ VA64). Adapted from data presented originally in Ref. [87]. Copyright 2023 Elsevier.

Vojinovic et al. used FTIR spectroscopy to detect the interactions between carbamazepine (CBM), Kollidon^®^ VA64, and Neusilin^®^ UFL2 (Figure 3). The shifting of the absorption bands characteristic of each functional group could be attributed to the interaction. FT-IR spectra of CBM were observed at 3463 cm^−1^ (–NH valence vibration), 1674 cm^−1^ (–CO-R vibration), 1593 cm^−1^, and 1605 cm^−1^ (range of –C=C– and –C=O vibration and –NH deformation). The shifting of the major absorption bands of CBM was observed in the ternary solid dispersion formulation. The sharp absorption peak of CBM at 3463 cm^−1^ changed to a broad absorption band at around 3460 cm^−1^, while the absorption band at 1674 cm^−1^ shifted to 1652 cm^−1^. The changes in these spectra were attributed to the hydrogen-bond interactions between the amide group of CBM and the carbonyl group of Kollidon^®^ VA64 and/or the silanol group of Neusilin^®^ UFL2 [87].

Shi et al. also reported the investigation of intermolecular interactions between telmisartan (TEL) and polymers in the ternary solid dispersion using FT-IR spectroscopy. The C=O stretch of carboxylic acid and the O-H stretch of TEL were observed at 1694.74 cm^−1^ and at 3443.49 cm^−1^, respectively, while the peaks of C=O stretch of the carbonyl from PVP K30 were observed at 1647.75 cm^−1^. The two peaks at 1636.00 cm^−1^ and 1733.64 cm^−1^ were observed in Soluplus due to the two C=O stretches of carboxylic acid and carbonyl. The O–H stretch of Soluplus was also observed at 3450.85 cm^−1^. In the binary system of TEL- PVP K30, the peak shift of the C=O stretch at 1694.74 cm^−1^ and the O–H stretch at 3443.49 cm^−1^ were observed. In contrast, in the physical mixture of TEL-PVP K30, there was no obvious change. This indicated that the formation of hydrogen bonds was observed in TEL-PVP K30 binary solid dispersions. Moreover, TEL and PVP K30 have a proton-donor group (–OH) and a proton-acceptor group (C=O), respectively. Thus, the hydrogen-bond interaction would occur between TEL and PVP K30. In the binary system of TEL and Soluplus, the C=O stretch of TEL gradually disappeared with the increasing concentrations of Soluplus. The movement of the O–H stretch of Soluplus was observed, suggesting that an interaction between the carbonyl of TEL and hydroxyl of Soluplus was found in the binary system. In the ternary solid dispersions of TEL-PVP K30-Soluplus, similar changes obtained in both binary systems were also observed. Shifts in the C=O stretch of TEL and the O–H stretch were observed, indicating that TEL interacted with both polymers in the ternary systems [88].

### 4.2. Differential Scanning Calorimetry (DSC)

DSC measures the difference in heat flow between a sample and a reference pan to measure the amount of heat absorbed or released during a phase transition of the sample. Exothermic or endothermic peaks accessed by DSC indicate different thermal events. In addition, DSC can be used as a TSD characterization technique, providing precise information on melting points, glass transition temperatures (*T_g_*), and energy changes associated with phase transitions, including crystallization and melting processes [89]. The miscibility of drugs and polymers is difficult to assess, but DSC is probably the most commonly used method. Surpassing the miscibility limit can result in the formation of drug-rich and polymer-rich phases, ultimately leading to amorphous–amorphous phase separation. This fundamental phenomenon underlies the characterization of drug–polymer miscibility. Typically, the presence of a solitary glass transition temperature (*T_g_*) signifies a miscible drug–polymer system, whereas the observation of two or more *T_g_* s indicates a two-phase system. Nevertheless, certain exceptions have been reported, including where a singular *T_g_* was observed in the phase-separated system while a dual *T_g_* configuration was observed in the supposedly miscible system [86,90].

DSC measurement was used by Mahboobian et al. (2022) to evaluate the ternary system of simvastatin (SIM), polyethylene glycol (PEG), and polyoxyl 40 stearate (Myrj 52). The melting endothermic peaks of Myrj 52 and PEG were observed at 42.3 °C and 60.7 °C, respectively, while the melting endothermic peak of SIM was observed at 137.9 °C. In the ternary physical mixtures (PMs), two endothermic peaks were observed, which contributed to Myrj 52 (first peak) and PEG (second peak). Both peaks were also detected in the ternary system, with a slight shift to lower temperatures. The absence of a SIM melting endothermic peak is probably related to the solubility of SIM in the melted carriers at low temperatures [91]. Fung and Suryanarayanan, (2018) also reported the *T_g_* of PVP-KTZ ASD, PVP-KTZ-SUC, PVP-KTZ-OXA, PVP-KTZ-TAR, and PVP-KTZ-CIT after evaluation using DSC measurement. PVP-KTZ ASD showed *T_g_* at 49.6 (±0.4) °C, with crystallization at 110.2 (±0.5) °C (Tc) and melting at 147.0 (±0.2) °C (Tm). The incorporation of TAR and CIT in PVP-KTZ ASD increased *T_g_* and decreased the crystallization tendency in ASD. The *T_g_* value of ternary ASD is similar to the *T_g_* value of the acid-KTZ co-amorphic system. PVP-KTZ-OXA ASD caused a marked increase in *T_g_* at the melting temperature of the crystallization phase (193.8 ± 0.3 °C). KTZ-PVP-SUC *T_g_* was 47.4 °C, and the melting temperature of the crystallized phase was 162.1 ± 0.1 °C. Thermal analysis showed that the addition of organic acids affected the solid-state properties of ASD, while PVP at 10% *w*/*w* had minimal effect on stability [80].

### 4.3. Powder X-ray Diffractometry (PXRD)

PXRD provides an improved overview of the crystalline properties of formulations, especially drug crystallinity. It is one of the most popular bulk techniques for analyzing the crystal structure of inorganic, organic, and polymeric materials. X-ray diffraction patterns of the drug crystal show sharp crystalline peaks, while the amorphous form of the drug indicates a halo pattern without the diffraction peaks of the drug crystal [84,86,92].

Prior study has demonstrated that the utilization of PXRD, in conjunction with pairwise distribution function calculations, enables the characterization of nearest-neighbor interactions and local structural modifications. These analyses provide valuable insights into the qualitative inference of drug–polymer miscibility. The polymer PHPMA was added to a binary solid dispersion composed of griseofulvin and HPMC AS. PHPMA caused an interaction between griseofulvin and HPMC AS. In this study, it was observed that griseofulvin remained in an amorphous state for more than 19 months when stored at 85% relative humidity. Conversely, the spray-dried form of griseofulvin showed a rapid transition to almost complete crystallization within 24 h under the same conditions. The technique employed in this study effectively identified the individual constituents of the crystalline forms present in TSD formulations [93].

Janssens et al. (2007) reported the PXRD patterns of itraconazole/TPGS 1000/PVPVA in ternary solid dispersions. X-ray diffraction analysis showed that all samples exhibited halo patterns, indicating that ternary amorphous systems had been successfully prepared with spray-drying [94]. Pardhi and Jain (2021) reported the PXRD pattern of the TSD system from bedaquiline fumarate (BDQN), poloxamer 188, and tocopheryl polyethylene glycol 1000 succinate (TPGS). BDQN showed characteristic diffraction patterns, which are in accordance with crystalline form III. The binary system showed sharp diffraction patterns, which were attributed to BDQN and poloxamer 188, indicating the presence of a crystalline form of the drug dispersed in the polymer matrix. The TSD system also showed sharp diffraction patterns of crystalline BDQN dispersed within the poloxamer 188 and TPGS matrix. These results indicate the presence of a micro-crystalline structure of BDQN within BSD and TSD formulations [95].

### 4.4. Polarizing Light Microscopy

The polarized light microscopy (PLM) technique was used to effectively detect amorphous drugs in ternary solid dispersions and to confirm the results obtained from DSC and PXRD analysis. This technique can also distinguish the crystal morphology of drug polymorphs to identify the possibility of polymorphic transitions. Vojinović et al. reported that the appearance of needle-shaped crystals in the micrographs of carbamazepine (CBM), Neusilin^®^ UFL2, and Kollidon^®^ VA64 system was observed, indicating that the polymorphic form of CBM occurred from monoclinic form III (prismatic-shaped) to triclinic form I (needle-shaped). However, the photomicrographs of needle-shaped crystals were not detected in the CBM and Neusilin^®^ UFL2 (binary) system, indicating that the polymorphic conversion from form III to form I did not occur. The results observed under PLM are in agreement with the PXRD pattern result. In contrast, visual observations obtained from PLM revealed that the identification of CBM polymorphic transitions using DSC provides misleading conclusions [87].

Gumaste et al. also reported that the results of the microscopic examination were in general agreement with those of PXRD. The ternary system of drug–polymer–surfactant films was investigated by PLM after storage for 1, 7, and 14 days. On day 1, there was no recrystallization of the drug and surfactant. However, the sample showed birefringence after 7- and 14-day storage. This could be due to the recrystallization of drugs and surfactants that occurred. This result was in agreement with the PXRD results. Thus, PLM can be used as a rapid and convenient tool to screen the miscibility of polymer–drug surfactants. However, although the sample exhibited birefringence due to the presence of drug crystals, surfactant crystals, or both, it was difficult to distinguish whether the birefringence was caused by the drug or the surfactant. Therefore, the investigation of DSC is necessary to identify any phase separation of drugs, polymers, and surfactants [96].

### 4.5. Thermogravimetric Analysis (TGA)

Thermogravimetric analysis (TGA) was used to determine the weight loss due to the degradation of each component in the ternary solid dispersion and the loss of residual solvents in the processed samples. This study utilized a thermal process to manufacture TSDs, and to determine the upper limit of processing temperature, the initiation of the thermal decomposition of the drug as a reference point was employed [84]. Tang et. al. reported the TGA thermograms of febuxostat (FEB), and each polymer was used for preparing ternary solid dispersions. In the TGA curves, FEB and poloxamer 188 showed degradation in the range of 175~260 °C and 300~420 °C, while PVP K30 was from 350 to 475 °C. In the sample of solid dispersion from FEB, the TGA thermograms indicated a three-step degradation. The first, second, and third mass losses were from 225 °C to 275 °C, 275~375 °C, and 375~460 °C, respectively. The shifting of the thermal degradation onset of FEB was observed from 175 °C to a higher temperature of 225 °C. This suggested that the thermal stability was enhanced by the ability of polymers to hinder the volatilization of FEB [97]. Amani et. al. reported that ezetimibe (EZ), PVP-K30, and poloxamer lost significant weight at 348 °C, 401 °C, and 453 °C, respectively. The residual solvent levels from the sample were observed between 3.8 and 5.8% in the solid dispersions, suggesting the efficient drying of the solid dispersion product [98]. The information on the thermal decomposition temperatures from each component is important, to avoid the risk of the API or excipients degrading during preparation by thermal methods such as melting and quenching [99].

### 4.6. Solid-State Nuclear Magnetic Resonance (ssNMR)

Solid-state NMR (ssNMR) is used as an adjunct to classical diffraction methods for characterizing solid-state materials. Bragg diffraction yields all long-range structures, while NMR provides information on aspects such as hydrogen atom positions and dynamics. The challenging accessibility resulting from diffraction properties can be overcome by employing solid-state NMR, which offers a significant advantage in determining the chemical shifts of materials with known structures. Furthermore, solid-state NMR allows for the acquisition of spectra corresponding to individual conformations, further enhancing its utility in these studies [86,100].

**Figure 4:**
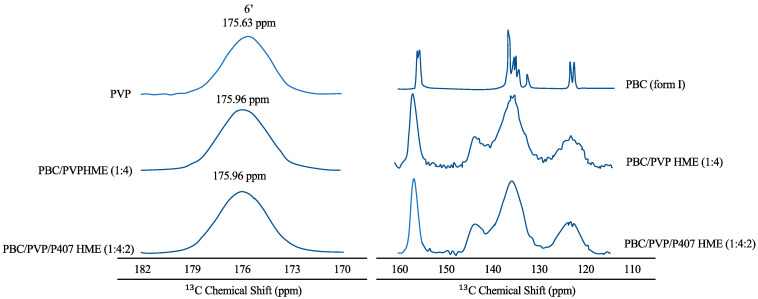
Solid-state ^13^C NMR spectra of each material. The arrows in the figure should be derived from the hydrogen-bond formation of PBC with PVP adapted from data presented originally in Ref [101]. Copyright 2023 Elsevier.

Hanada et al. (2023) reported that PXRD data confirming the amorphous form of probucol (PBC) in the ternary hot-melt extrusion (HME) were validated by solid-state NMR spectroscopy, as shown in Figure 4 [101]. The peaks of the aromatic ring carbon of PBC and PVP were found in the solid-state ^13^C NMR spectra of PBC in 115–160 and 170–182 ppm areas [102,103]. Furthermore, the sharp peaks of the PBC crystal entirely disappeared in the spectra of PBC/PVP HME (1:4) and PBC/PVP/P407 HME (1:4:2). The distribution of isotropic chemical shifts, reflecting the various conformations of the amorphous material, also explained the broad peaks. Peaks at 144 ppm (shown by arrows) were detected for both PBC/PVP and PBC/PVP/P407 HMEs, indicating that PBC established hydrogen bonds with PVP even when poloxamer 407 (P407) was present. A downfield shift of the PVP carbonyl carbon signal was also seen for PBC/PVP HME (175.96 ppm) and PBC/PVP/P407 HME (175.63 ppm), indicating the creation of a hydrogen bond between PBC and PVP. In contrast, the peaks of P407 in PBC/PVP/P407 matched closely with semi-crystalline P407, indicating the absence of interaction between PBC and P407 in the ternary HME [104]. Therefore, the hydrogen bond between PBC and PVP is primarily responsible for the stability of amorphous PBC in the ternary HME [105].

Ziaee et al. (2017) also used ^13^C solid-state NMR spectroscopy to assess the molecular state of ibuprofen in the TSD system. Due to the overlapping peak between the excipients and ibuprofen, the program DMfit was used to simulate the spectra. Two samples of TSD systems from ibuprofen were evaluated. In the sample of TSD 1, sharp peaks like those observed in the ^13^C NMR spectrum of ibuprofen were observed, while these are notably absent in the ^13^C NMR spectrum of the sample of TSD 2. This indicated that crystalline ibuprofen is present in the sample of TSD 1 and absent in the sample of TSD 2, which is consistent with the powder X-ray diffraction data. The broad peaks with chemical shift(s) in the ^13^C NMR spectra were observed in the sample of TSD 1 and the TSD 2 samples 1 and 4, indicating the presence of amorphous ibuprofen. The sharp peak at 183 ppm indicates that the crystalline ibuprofen in the sample of TSD 1 is not interacting with the excipients. In contrast, a shift in resonance from δiso = 181 ppm from the ibuprofen crystal to 178 ppm in the TSD system was attributed to hydrogen-bond interaction between the carboxyl group in ibuprofen and PVP. The shift in carboxyl carbon resonance frequency was also observed from δiso = 183 to 177 or 178 ppm, which was attributed to hydrogen-bond formation between the carboxyl group in ibuprofen and carbonyl groups in the excipients [106].

### 4.7. Dielectric Spectroscopy

The α-relaxation time is a measure of the molecular mobility of a sample determined by dielectric spectroscopy. Li et al. (2022) reported the temperature dependence of α-relaxation time (τα), in the supercooled state, of amorphous APIs, SMZ-TMP co-amorphous systems, and ternary ASDs [79]. These data supported the conclusion of DSC from the formation of a single amorphous phase. Meanwhile, the system of SMZ-TMP (5:1) with a co-amorphous structure shows about three more orders of magnitude reduction in molecular mobility than the amorphous SMZ. In addition to immobilizing the system, EDE has led to an additional decrease, of two orders of magnitude, in molecular mobility. For the SMZ-TMP (1:5) system, the co-amorphization caused around an increase of one order of magnitude in α-relaxation time compared to amorphous TMP. The addition of PAA then increased the α-relaxation time by four orders of magnitude. The preceding discussion presented a rather generalized perspective that did not apply to the entire range of temperatures concerning both the 5:1 and 1:5 ratios. In this case, drug–drug and drug–polymer interactions ‘immobilized’ the drugs, but the general ranking in terms of molecular mobility was ternary ASDs < co-amorphous systems < amorphous APIs.

Fung et al. reported that the addition of PVP (10% *w*/*w*) caused an insignificant increase in relaxation time. The effects of CIT and OXA were equivalent and led to an approximately twofold increase in relaxation time when compared to KTZ-PVP. The effect of incorporating TAR into PVP-KTZ ASDs caused the greatest decrease in molecular mobility. In addition, the KTZ-PVP-SUC ASD is similar to KTZ-PVP in terms of mobility. The KTZ acid co-amorphous system and the ternary ASD are brittle, with D values ranging from 6.0 to 10.9. The relaxation time of KTZ-PVP-CIT was longer by more than two orders of magnitude than amorphous KTZ-CIT. In the OXA and TAR systems, the mobility of the ternary ASD is comparable to the equivalent acidic KTZ system. In KTZ-PVP-SUC, the mobility is comparable to PVP-KTZ and KTZ-SUC. This was a rare case where two binary mixtures and a ternary system had similar mobility properties, specifically at higher temperatures [80].

### 4.8. Scanning Electron Microscopy (SEM)

SEM was used to examine the particle size and shape of the ternary ASD. Fung et al. reported that the addition of 10% *w*/*w* PVP to KTZ-PVP results in dispersion with a *T_g_* of 49.6. After passing through the exit of the spray dryer, the particles were subjected to a transformation, adopting a spongy rubber-like shape. Consequently, the PVP-KTZ ASD particles seemed to aggregate initially before ultimately forming genuine agglomerations. The integration of SUC in the PVP-KTZ ASD was not effective in lowering the molecular mobility of the system, which explained the high particle size of PVP-KTZ and PVP-KTZ-SUC. However, the system’s molecular mobility significantly decreased with the addition of CIT, OXA, and TAR. These systems often have little molecular mobility and were in a glass-like condition in the spray-dryer cyclone. These particles were tiny and spherical, according to SEM. The increased surface area of the spray-dried particles was used to partially explain the quick disintegration in the CIT, OXA, and TAR systems. Additionally, the ternary systems’ particle size and shape resemble the co-amorphous systems [80].

The summary of some useful techniques for the characterization of the TSD system in the solid state can be seen in Table 6.

## 5. Characterization of Ternary Solid Dispersion after Being Dispersed in the Water

### 5.1. Dynamic Light Scattering (DLS) Measurement

DLS is an established and precise measurement technique to analyze the particle size distribution of the TSD system after dispersal into water. Zhao et al. (2021) studied DLS analysis to determine the particle size distribution of ternary solid dispersions prepared by the spray-drying method after being dispersed in distilled water (protocol 1) or a solution containing HPMC and sodium dodecyl sulphate (SDS) (protocol 2). The HPMC and SDS concentrations of the solution were adjusted in protocol 2 to achieve the total added amount of PBC, HPMC, and SDS in each final TSD suspension. The formation of tiny nanoparticles with a mean volume diameter (MV) below 30 nm was observed in suspensions of protocol 1. The samples showed polydispersity index (PDI) values less than 0.3, suggesting the monodispersing of drug nanoparticles [107]. The TSD suspensions with low weight ratios of SDS and HPMC in the TSD system showed enlarged MVs and PDIs. Therefore, high weight ratios of SDS and HPMC in the TSD system were desirable for producing the drug nanoparticles. The MVs and PDIs in protocol 2 were generally smaller than in protocol 1. The pre-dissolved HPMC and SDS can decrease the particle size or inhibit aggregation/agglomeration [108].

Pardhi and Jain (2021) reported a DLS analysis of the TSD system from BDQN, poloxamer 188, and TPGS. In the binary system, the average size was found at 567 nm, with a larger PDI value of 0.77, indicating the more extensive size range of particles. However, the TSD system showed a good PDI value of 0.4 (in comparison to the binary solid dispersion (BSD)) with an average size of 792.7 nm, indicating good stability in a dispersed state. The accelerated stability sample of the TSD system exhibited size and PDI values of 809.2 nm and 0.41, respectively, indicating variable size distribution compared to the TSD not exposed to accelerated conditions. These results declare that the presence of TPGS as a surfactant can induce good size distribution, meaning it is nearly monodispersed in comparison to the BSD [95].

### 5.2. TEM and Cryo-TEM Measurements

Cryogenic transmission electron microscopy (Cryo-TEM) is used to observe changes in the size and shape of nanoparticles to reflect the nanometer-scale morphology of each nanoparticle in the suspended state [104]. Zhao et al. (2021) used this method to observe the nanometer-scaled morphologies of the particles in the SD suspension. In protocol 1 (the TSD was dispersed in distilled water), the PBC nanoparticles were spherical, with a mean number diameter (MN) of approximately 20 nm. The spherical shape of PBC suggested its supercooled liquid state. This could be due to the preparation temperature of the TSD suspension being 25 °C, which is slightly higher than the glass transition temperature (*T_g_*) of amorphous PBC (23 °C). In the other TSD system with different SDS amounts, a difference in shape was observed. The MN of PBC gradually increased with the decreasing weight ratios of HPMC. On the other hand, in the other TSD suspensions with low weight ratios of HPMC, some particles formed coalesced agglomerates. The agglomeration became more intensive and formed irregular structures with hundreds of nanometers, leading to the formation of needle-like nanocrystals with particle lengths of over 200 nm. These findings suggested that the increasing amount of HPMC in the TSD system can suppress the mean particle size of the spherical PBC amorphous nanoparticles. Moreover, the addition of SDS can significantly suppress the agglomeration and crystallization of PBC [108].

TEM imaging was also performed by Zhao et al. (2019) to determine morphology in micrometer-sized deposits. In ground-mixture (GM) deposits, micrometer-sized hexagonal and crystal rods were observed, corresponding to the morphology of micrometer-sized crystals. Cryo-TEM and TEM images showed that the micrometer-sized deposits observed were not secondary particles. Consequently, no intermediate structures, indicative of aggregation or agglomeration, were formed throughout the entire storage period. These findings strongly suggested that the formation of micrometer-sized crystals occurred through the growth-phase-mediated transformation of needle-like nanocrystals. Spherical nanoparticles with diameters ranging from 50 to 100 nm were also found immediately after production in the HME colloidal dispersion (1:4:2). The cryo-TEM image demonstrated that there was no significant variation in size and shape between the PBC particles before or after 14 days of storage, indicating no substantial agglomeration or crystallization. Therefore, the storage stability of the HME colloidal dispersion (1:4:2) was confirmed [109].

### 5.3. Atomic Force Microscopy (AFM) Measurements

The evaluation of spherical nanoparticles of TSDs after being dispersed in the water was performed with the AFM tapping method [109]. Circular particles were observed on each suspension, according to cryo-TEM images. The cross-sectional profile showed nanoparticles with a height of 20−30 nm and a width of 50−70 nm, except for a relatively large number. The width of the nanoparticles was larger due to the convolution effect of the AFM probe. Furthermore, the width of the nanoparticles was calculated using the tip shape and the average height was 48−63 nm, which was different from the width of 50−70 nm. The nanoparticles must have a morphology to maintain a spherical shape during AFM imaging. After taking altitude images using the tap mode, AFM distance–force curve measurements were performed over 50 particles after the aqueous dispersion, using the contact mode to analyze mechanical characteristics.

The distance between the point of contact and substrate for all distance–force curves for spray-dried sample (SPD) and ground-mixture (GM) suspensions was consistent with the height of the nanoparticles shown in the cross-sectional profile. These results confirmed that distance–force curves were recorded for the centers of the nanoparticles. In the distance–force curves of the selected SPD nanoparticles, the approximation curves were very low despite the difference in particle size. This discovery suggested that nanoparticles in SPD suspensions formed softer structures. The force increased almost linearly for the movement from the point of contact to the substrate. Furthermore, the nanoparticles in SPD suspensions were subjected to elasticity deformation during compression. Stretching was induced by tip retraction and the maximum adhesive strength was less than 0.2 nN, suggesting a very soft nanoparticle structure. To ensure proper positioning, the tip should have come into contact with the nanoparticles instead of sliding to the side of the sample. This was supported by the observation of prolonged continuous dissipation distances on the retractation curve.

Nanoparticles in GM suspensions showed different AFM distance–force curves than SPD suspensions. The suspension curve approach of GM was unusual, initially showing a gradual slope after the point of contact, with force then increasing until the tip reached the substrate. In addition, the gradual slope was caused by a slight deformation caused by compression. This effect showed the lower elasticity and higher stiffness of nanoparticles in GM suspensions compared to particles in SPD suspensions. A stronger maximum adhesion above 0.5 nN also indicated the more rigid structure of nanoparticles in the GM suspension [109].

### 5.4. ^1^H Solution-State NMR

It is possible to use ^1^H solution-state NMR spectroscopy to determine the hydrogen-bonding interactions between the compounds and the polymer in the solution phase. Borde et al. (2021) reported ^1^H solution-state NMR spectroscopy analysis to confirm the interaction of in-dole2-carboxamide derivative (North 2) in the TSD system after dissolving the dispersions in deuterated chloroform. The upfield shift means that the proton may be involved in the interactions, resulting in a shift of the peak to the shielded region in the NMR. The upfield chemical shifts (having a positive delta) in the peak of indole N–H and amide N–H of North 2 were observed in the TSD system, indicating that these protons are involved in the interactions. Meanwhile, in the aliphatic and aromatic regions, there were no peak shifts observed, suggesting that these protons are not involved in the bonding interactions [110].

Zhang et al. (2020) also used ^1^H solution-state NMR spectroscopy to investigate the dissolving behavior of Olmesartan medoxomil (OM) in ternary solid dispersions. The ternary solid dispersion revealed the significant decomposition of OM in the D_2_O solution due to its natural metabolism with the breakage of the ester bond. The decomposition of OM was detected at around 70% in the TSD system. In contrast, the physical mixture of OM + hydroxypropyl-βcyclodextrin (HP-β-CD) + N-methyl-D-glucamine (MG) (molar ratio 1:1:1) showed a ~25% decomposition of product after 2 h of solvation and ~60% after 70 h. This result indicated that the TSD system of OM promoted the breakdown of the ester to Olmesartan molecules in water solution. The chemical shifts of OM were observed in the TSD system due to intermolecular hydrogen-bond formation between various functional groups of OM and OH groups of MG [111].

### 5.5. Zeta Potential

Hanada et al. evaluated the zeta potential of the TSD system in the dissolution medium. The zeta potential of amorphous PBC nanoparticles in the HME colloidal dispersion (1:4:2) was determined to be −11.6 mV to examine dispersion stability. The absolute value of the amorphous PBC nanoparticles (−11.6 mV) was lower than the result reported for PBC particles (−41.0 mV), indicating the adsorption of P407. The steric repulsion of the P407 coating on the particle interface resulted in the strong dispersion stability of the amorphous PBC nanoparticles [101]. Pardhi, et al. (2021) also reported that the monodispersed particles of the TSD system with an average zeta potential distribution of 23.1 mV were observed, indicating good stability in the dispersed state. The accelerated stability sample of the TSD demonstrated a surface zeta potential of 17.3 mV, suggesting that the variable size distribution of the TSD was not significantly changed after exposure to accelerated conditions. The reduction in zeta potential after exposure to accelerated conditions was attributed to the dispersed TSD in water and the good stability zone of colloidal systems [98].

The summary of some useful techniques for the characterization of the TSD system after being dispersed in water can be seen in Table 7.

## 6. Dissolution Study of Ternary Solid Dispersion Systems

### 6.1. Dissolution Mechanisms

TSD formulations evolved faster than binary systems amid growing concerns about the solubilization of insoluble drugs. However, the rational formulation design of the TSD system and the molecular mechanism of its dissolution were under development. The molecular structure was characterized by differential scanning calorimetry (DSC), infrared absorption spectroscopy (IR), X-ray diffraction (XRD), and scanning electron microscopy (SEM). Molecular dissolution mechanisms in BSDs and TSDs were determined by a combination of experiments and molecular modeling techniques [112].

### 6.2. Factors Affecting Dissolution

A solid dispersion of griseofulvin containing polyethylene glycol (PEG) showed that the dissolution rate was dependent on the particle size and the dissolution rate was controlled by the drug. Furthermore, the interaction between indomethacin and the carrier ethylcellulose occurred when the pH of the medium was low, slowing the release of indomethacin [113].

A major challenge during the dissolution of solid dispersions was to prevent drug precipitation. After the dissolution of the solid dispersion, micro- or nanoparticles are generated, subsequently dispersing within the medium. However, since supersaturation is achieved, these particles possess a tendency to precipitate into the dissolved matrix or bulk solution. The involvement of drug–polymer interactions was proposed to assume a significant role in suppressing drug precipitation and effectively stabilizing the amorphous state throughout the process of solid dispersion dissolution [60].

### 6.3. Effects of Physicochemical Properties of Drug

The effect of cooling technology on the physicochemical properties of drugs, such as manidipine hydrochloride (MDP), is an area of investigation. In this study, the solubility of MDP was examined using a thermal stress device (ThSD). To prepare the ThSD, MDP was subjected to the melting process, and the resulting viscous mass was solidified by cooling in an ice bath or with the use of liquid nitrogen. Subsequently, the dried mass was ground into a powder and stored at −20 °C until further analysis. The physicochemical properties of the solid dispersion powders were characterized through solubility tests, powder X-ray diffractometry (PXRD), and differential scanning calorimetry. The findings indicated that the solidification of the mass using liquid nitrogen and an ice bath led to a significant increase in the solubility of MDP when compared to native MDP. Specifically, the use of liquid nitrogen cooling was observed to provide higher solubility for amorphous products than ice baths. [114].

### 6.4. Effects of Preparation Method

Numerous preparation methods have been established and developed to produce solid dispersions, including co-milling, melting, solvent evaporation, and co-precipitation processes. Previous studies showed that the choice of manufacturing method has a significant impact on physicochemical behavior, with results such as amorphization, enthalpy relaxation, and the recrystallization of the resulting system, leading to different performances of the final product. Therefore, the choice of the manufacturing process and the strategy for selecting each class of solid dispersions are important [115,116].

### 6.5. Effect of Co-Formers

Co-former solubility and the strength of the drug–co-former interaction affect the dissolution rate. In studies of naproxen-tryptophan-proline and naproxen-arginine/proline ternary amorphous mixtures, naproxen was stabilized by co-amorphization with high levels of *T_g_* tryptophan or by salt formation with arginine [117]. The dissolution result profiles of KTZ, KTZ-PVP, and KTZ-PVP acid TSDs have also been reported. PVP functions as a crystallization inhibitor, but the addition of PVP at 10% *w*/*w* resulted in a slight increase in the dissolution of KTZ. Meanwhile, the addition of organic acids (KTZ-acid molar ratio of 1:1) into the PVP-KTZ dispersion increased the solubility of KTZ. Among the four acids, the greatest solubility enhancer was OXA, with a ~5-fold increase relative to the binary KTZ-PVP ASD. Furthermore, the increase in solubility can be sorted as follows: OXA > TAR ≈ CIT > SUC. The acid plays the role of lowering the pH of the diffusion layer around dissolving solids, increasing the solubility of KTZ. Therefore, the effect appears to become more pronounced as the strength of the acid increases. In the KTZ-PVP-TAR and KTZ-PVP-OXA systems, a significant portion of KTZ, approximately 85%, readily dissolves into the solution. This efficient dissolution is primarily attributed to the presence of excipients, which effectively facilitate the rapid dissolution of most of the drug [82]. Ueda et al. also reported the intrinsic dissolution rate (IDR) of the crystalline CBM and the co-amorphous samples. The intrinsic dissolution characteristics of the co-amorphous CBM-CA (1:1), CBM-CA-ARG (1:1:1), CBM-CA-ARG (1:1:2), and CBM-CA-ARG (1:1:3) are in crystalline form. Crystalline CBM had the lowest IDR of any sample tested, at 0.025 mg/mL/min, and by creating a co-amorphous system with CA (0.055 mg/mL/min), the IDR was enhanced. The IDR was better in the ternary co-amorphous CBM-CA-ARG systems (0.067, 0.082, and 0.085 mg/mL/min at molar ratios of 1:1:1, 1:1:2, and 1:1:3, respectively). In previous studies, it was reported that a drug’s rate of dissolution in a co-amorphous system may be accelerated by the addition of a highly water-soluble co-former [117]. To improve the dissolving pace of CBM, the ratio of hydrophilic salt co-former in the co-amorphous form should be increased. IDR values of co-amorphous CBM-CA-ARG (1:1:2) and CBM-CA-ARG (1:1:3) are almost the same, meaning the addition of CA-ARG (1:2) is sufficient to increase IDR CBM [78].

### 6.6. Effect of Polymers

To improve the dissolving profile, hydrophilic polymers including polyvinylpyrrolidone, polyethylene glycol, hydroxypropyl methylcellulose, and poloxamer were used to molecularly disperse the weakly water-soluble medicines in solid dispersions (SDs). The choice of an ideal polymer was essential for the development of efficient SD formulations, to inhibit the recrystallization of amorphous medicines in supersaturation conditions [118]. Furthermore, Zoeller et. al. reported the dissolution profile of the glyburide in ASDs (drug–polymer) and TSDs (drug–polymer–polymer). The binary system of glyburide showed rapid release and around 55% of the dose dissolved within 30 min. In contrast, the ternary system improved the dissolution performance of glyburide compared to the binary system. The amount of drug release was around 80%, and the presence of polymer increased the wettability of the formulation due to its hygroscopicity, leading to a faster or instant drug release [61].

### 6.7. Effect of Surfactants

Surfactants can be added to improve the dissolution properties of a drug [119]. They change the rate of precipitation (drug delivery in solution), increase membrane permeability, and affect the integrity of membranes, which can all affect how quickly solid dosage forms dissolve and disintegrate. Consequently, this process enables the formation of micelles that effectively encapsulate and distribute hydrophobic drugs within their core, leading to enhanced drug solubility. The use of TSDs proves to be highly effective in promoting miscibility, preventing the growth of new crystals, and creating homogeneous dispersions. To reduce the interfacial tension between the solvent and SD powder, surfactants were employed. The anionic surfactant, SDS, exhibited superior solubilization ability compared to non-surfactant TWE systems. Even though several surfactants have been shown to improve the dissolution profiles of drugs, SDS demonstrated an enhanced performance in solubilizing the drugs [115]. Gamal et al. investigated the effect of Pluronic F68 on the dissolution profile of itraconazole in the ASD system. The dissolution of itraconazole (ITZ) in the ASD system with HPMC was enhanced compared to ITZ crystals. However, the profile showed a slow release, with drug dissolution reaching 72% after 2 h. The TSD of ITZ prepared by melt extrusion showed a significant increase in drug dissolution. The increase in drug dissolution with the presence of Pluronic F68 was attributed to the solubilizing effect of the surfactant [44].

## 7. Physical Stability

### 7.1. Factors Affecting Stability

The formation of physically stable ternary amorphous systems by solid-state dispersion methods using optimized ball-milling techniques was an alternative for improving drug solubility and stability. This was achieved to improve the dissolution rate of some pharmaceuticals, such as probucol, gliclazide, and naproxen. The pharmacokinetic properties and in vivo bioavailability were significantly improved compared to the pure drug molecule. Furthermore, the dissolution rate of ibuprofen was significantly increased in the presence of PVP and complexation by βCD. This was due to various mechanisms (intermolecular interactions, carrier synergy, anti-plasticizing effect, increased hydrophilicity, decreased particle size, and accretion to βCD cavities) that promote the stabilization of amorphous ibuprofen under stress conditions [120].

### 7.2. Effects of Physicochemical Properties of Drugs

One study shows the physical stability of felodipine solid dispersions under different storage conditions. For the low-viscosity hydroxypropylcellulose (HPC-SSL)-felodipine solid dispersion, phase separation and recrystallization occurred at low and high storage temperatures. It is important to mention that some semi-crystalline supports, such as PEG and poloxamers, were converted to amorphous forms, resulting in mixed systems. The production of a solid oxeglitazar dispersion through the utilization of the spray-freezing technique showed an intriguing discovery. This phenomenon was elucidated by the establishment of intermolecular interactions, primarily involving H-bonds, between the active pharmaceutical ingredient (API) and the supporting material. Therefore, the physical state of both components may be influenced [116]. Li et al. reported the physical stability of TSD and co-amorphous systems. For the amorphous SMZ-TMP system (5:1), crystalline SMZ and the salt appeared at 80 °C and 110 °C. In the co-amorphous SMZ-TMP system (1:5), TMP and the salt crystallized at 80 °C and 100 °C. Furthermore, the addition of EDE and PAA significantly improved physical stability. For the PAA ASD, the crystalline phases of TMP and SMZ-TMP salts appeared at 140 °C and 150 °C, respectively. The ternary EDE-ASD showed several low-intensity peaks around 150 °C, but the crystallized phases were not identified. At 40 °C/75% RH, ternary ASDs were stable for at least 6 weeks but after storage for 24 h, the amorphous SMZ and the SMZ-TMP co-amorphous systems were crystallized [79].

### 7.3. Effects of Preparation Method

The physical state of solid dispersions, particularly their ability to “amorphize” and “crystallize,” was affected by the preparation and storage conditions, resulting in stability issues. Therefore, it was crucial to understand the physical state of the drug and the polymer to achieve the optimal stability and solubility of solid dispersions [116].

Recent studies have investigated the efficiency of the co-grinding process compared to other processing techniques such as melting, spray-drying, and co-precipitation to analyze the effect on solid dispersion efficiency. The results demonstrated that solid dispersions prepared using the co-milling method exhibited higher heterogeneity, fewer drug–polymer interactions, and lower physical stability compared to other methods [116].

## 8. Mechanism Drug Release of Ternary Solid Dispersion Systems

The solid dispersion of the active ingredient in three separate components of a drug delivery system consisting of three main components—namely drug, drug carrier, and excipients—in a solid state is referred to as a ternary solid dispersion (TSD). The addition of a third ingredient, such as a different polymer, surfactant, excipient, or compatible medication, improved the formulation’s solubility and ultimately enhanced its stability.

**Figure 5:**
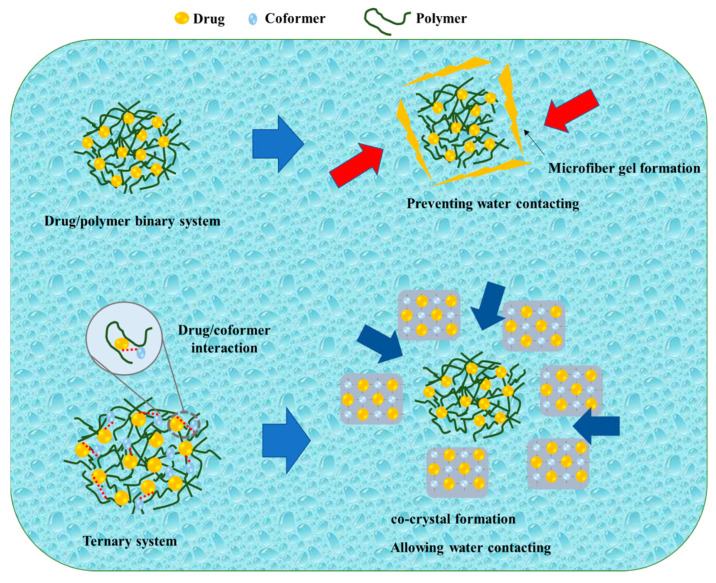
A schematic illustration of a binary system and a ternary system, adapted from data presented originally in Ref. [81]. Copyright 2023 Elsevier. 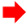: Preventing the water contacting to amorphous drug, 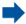: Allowing the water contacting to amorphous drug.

Kosaka et al. reported on the mechanism of the TSD system after its dispersion in water, as shown in Figure 5. During the initial stages of the dissolution test, the amorphous drug rapidly crystallized into an alpha-crystal state. Even though there was a temporary improvement due to amorphization, the drug concentration remained constant around the solubility of the alpha-crystal state. In contrast, the binary solid dispersion consisting of the drug and polymer exhibited a high drug concentration at the beginning of the dissolution test, which decreased over time. According to Heng et al., the amorphous drug aggregated into a microfiber-like gel, impeding drug dissolution. The amorphous drug existed in a supercooled liquid state, becoming highly viscous when in contact with the dissolving medium. Consequently, drug molecules adhered to form a viscous aggregate self-assembled into a three-dimensional network to prevent the amorphous drug from coming into contact with the dissolving liquid, resulting in a decrease in concentration. In the current investigation, when the IMC/PVP solid dispersion came into contact with the dissolving medium, the presence of hydrogen bonding between the drug and polymer facilitated rapid dissolution at the beginning of the test. As the dissolution test progressed, a phase rich in amorphous drugs, formed through phase separation, gradually transformed into a microfiber-like gel on the surface of the dispersed particles in the dissolving medium. The three-dimensional network of the amorphous drug microfiber-like gel prevented the ASD system from making contact with the dissolving media. Even though most of the drug remained in the amorphous state inside the particle, the microfiber-gel precipitation on the surface caused a steady drop in drug concentration. The TSD system maintained a high drug concentration for a relatively extended duration and the disintegration occurred at a slower rate compared to the binary SD. Since the hydrogen link between the drug and co-former was formed in the TSD, the binary system (cocrystal) precipitated at the particle surface at the beginning of the dissolution test within 1 h. Furthermore, cocrystal development on the surface prevented the initial drug dissolution from the TSD system. On the surface, the system had intermediate spaces, granting access to the dissolving liquid. Therefore, the particles’ internal ASD (drug/polymer) dissolved into the dissolution medium, maintaining a high drug supersaturation [81]. This study suggested that the hydrogen bond between the drug and co-former in the cocrystal system remained in the TSD system (after polymer addition), leading to significant differences in the dissolution profiles between the drug/polymer binary system and the TSD system caused by the hydrogen bond between the drug and co-former.

**Figure 6:**
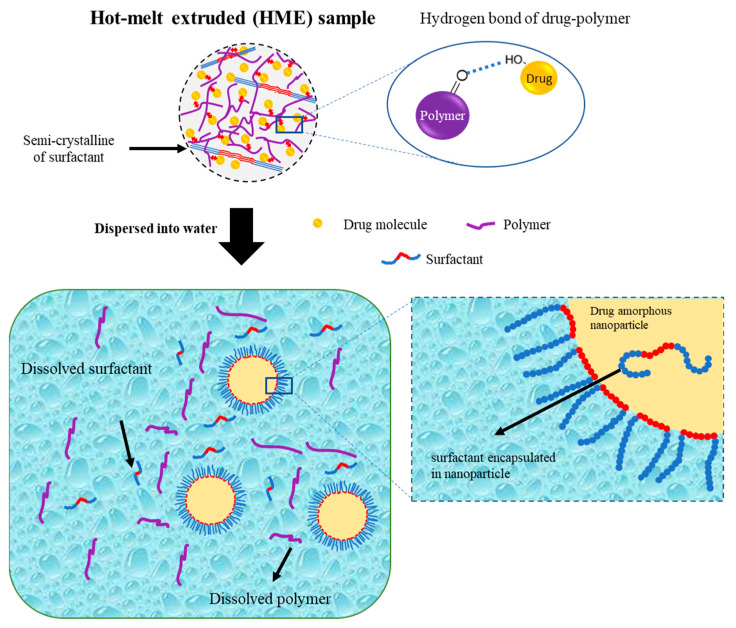
Schematic illustration of a ternary solid dispersion prepared by HME, adapted from data presented originally in Ref. [101]. Copyright 2023 Elsevier.

Hanada et al. also reported a schematic illustration of the colloidal dispersion formation of amorphous drug nanoparticles from the TSD system prepared by the HME method, as shown in Figure 6. The hydrogen bond formed by the hydroxyl proton of the drug and the carbonyl oxygen of the polymer was primarily responsible for stabilizing the amorphous drug in the TSD. The polymer matrix contained drug molecules and drug-rich regions. Meanwhile, most of the surfactant in the TSD system was semi-crystalline, with small interactions with the drug. Amorphous–amorphous phase separation caused by water contact also resulted in the formation of nanoscale drug-rich regions in ASDs. Drug-rich domains were released into the water due to the water-soluble polymer and surfactant, resulting in a colloidal dispersion of amorphous drug nanoparticles. The amorphous state of the drug in the TSD and colloidal dispersions was stabilized in part by the polymer and surfactant. Moreover, the presence of the polymer prevented drug recrystallization upon contact with water due to the existence of hydrogen bonding. Surfactants were more effective in stabilizing the amorphous state of the drug in colloidal dispersions and were capable of adsorbing at the interface of drug nanoparticles when dispersed in water. This ability provided stabilization to the drug’s amorphous form by preventing direct exposure to water. The polymer acted as a conformational barrier when it encountered water, impeding the further aggregation of individual drug-rich domains. In the presence of water, surfactants were adsorbed onto the interface, resulting in the formation of a U-shaped structure. This structure consisted of hydrophilic and hydrophobic polyethylene oxide chains extending towards the aqueous phase and nanoparticles. Furthermore, surfactants should cover the interface of the drug-rich regions and prevent particle aggregation after contact with water. The U-shaped structure created by the molecules covering the particle interface increased the colloidal nanoparticles’ storage durability by preventing drug crystallization and particle agglomeration. The inclusion of additional molecules within the nanoparticles played a crucial role in stabilizing the amorphous form of the drug during storage through the reduction in the thermodynamic activity of the supercooled liquid drug [101]. Therefore, the preparation of a ternary solid dispersion by applying hot-melt extrusion can form a stable amorphous drug after being dispersed in the water. The polymer can reduce the particle size and stabilize the amorphous drug, while the surfactant can maintain the amorphous drug nanoparticle size.

**Figure 7:**
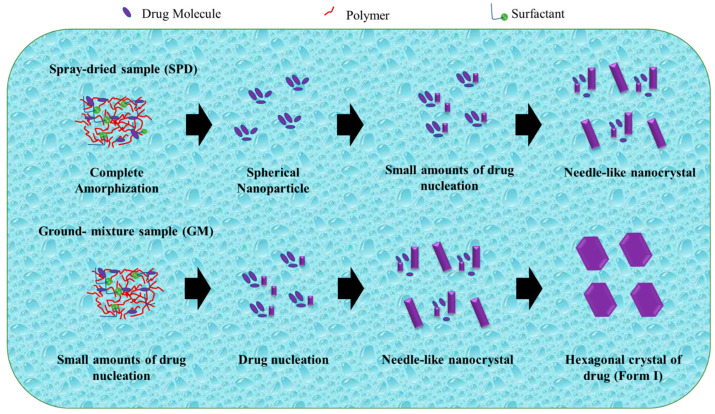
A schematic illustration of the evolution from a ternary solid dispersion after dispersion in water. Adapted from data presented originally in Ref. [109]. Copyright 2023 ACS Publications.

Zhao et al. studied the mechanism of TSD systems after dispersal in water, as shown in Figure 7. Exposure to water can induce amorphous phase separation in a solid dispersion, leading to the formation of nanosized drug domains. This phenomenon holds promise as a potential method for nanoparticle production. In addition, amorphous drug domains are instantly liberated as drug nanoparticles move into the bulk solution. The stability and size are significantly influenced by the dispersibility of the manufactured nanoparticles as well as the dissolving rate of the medication. The mechanism of nanoparticle production explains the survival of a small number of drug nuclei or drug-rich domains, which retain the short-range order of the initial crystal form, within spherical drug nanoparticles following their dispersion in water [109].

The drug was totally amorphized and blended uniformly with polymer and surfactant in the TSD system prepared by the spray-drying technique. After dissemination in water, drug nucleation and crystal formation were effectively prevented or postponed, and the amorphous state of the drug was sustained. During the storage of spherical nanoparticles in water, the progressive increase in particle size can be attributed to Ostwald ripening. Ostwald ripening occurs as a result of changes in local solubility based on particle size, leading to the transportation of drug molecules from smaller to larger particles. Furthermore, the smaller particles dissolve as the large particle size increases. After storage for 12 days and 1 day, the nanoparticles show an increase in particle size and a decrease in PDI. In addition, the distribution of the spherical nanoparticles narrowed as the particle size gradually increased. After 1 day of storage, the PDI values (about 0.090.12) of freshly prepared samples declined to roughly 0.07 for the SPD and GM(I) suspensions. The PDI values in the SPD samples changed more slowly, from 0.07 to 0.05 for 1 day and 12 days of storage. The difference in solubility between smaller and larger particles was the driving force behind Ostwald ripening [109].

The amorphous drug within the spherical nanoparticles in water tends to crystallize to reduce its elevated Gibbs free energy. Despite the partial crystallization of the drugs, the nanoparticles maintain their spherical shape. Further crystallization transforms the morphology from spherical to a distinct crystal structure. In a study conducted by Egami et al., the utilization of AFM force–distance curves enabled the elucidation of the gradual crystallization process of the drug within the nanoparticles. Even though it has been described for inorganic compounds, direct morphological study of the nucleation pattern of amorphous nanoparticles is uncommon for organic compounds. In contrast, the co-grinding approach facilitates the amorphization of the drug through a gradual reduction in the crystal size through mechanical force. This approach retains the presence of extremely small amounts of drug-rich domains exhibiting short-range order, which closely resemble the original crystal shape despite most of the drug existing in an amorphous state [109]. The preparation method of ternary solid dispersions can affect the stability of the amorphous drugs in the water, which is very important for the bioavailability of drugs. Therefore, studies to elucidate the molecular states of ternary solid dispersions after being dispersed in water are necessary to produce a ternary solid dispersion formulation with a stable amorphous drug in water.

## 9. The Mechanism of Stability Enhancement of Ternary Solid Dispersion Systems

**Figure 8:**
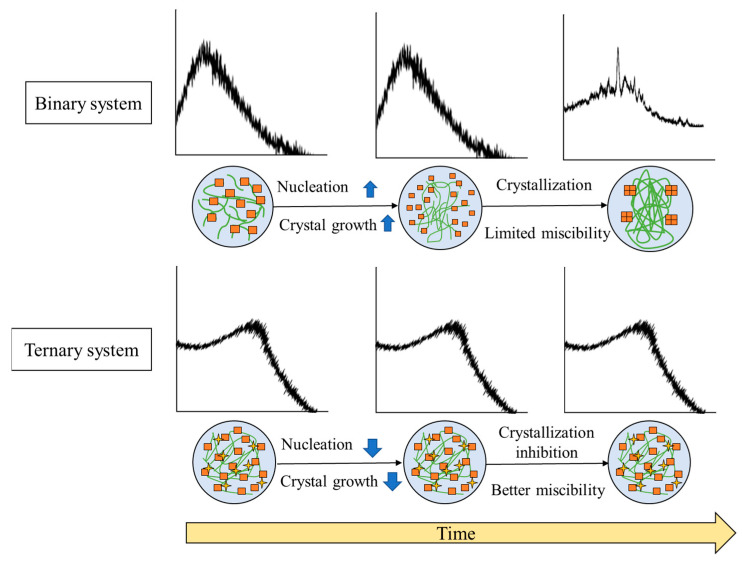
Illustrates the general mechanism of the stability enhancement of TSDs compared to binary systems. Adapted from data presented originally in Ref. [35]. Copyright 2023 Taylor and Francis.

The general mechanism of stability enhancement of amorphous drugs in TSD systems can be seen in Figure 8.

Precipitation occurred in the binary system after storage in the presence of high temperature or humidity, leading to an instability of formulation caused by the recrystallization of amorphous drugs. These phenomena are due to the dominance of drug–drug interactions over drug–polymer interactions. In contrast, the inclusion of a third component serves as a bridging component acting as a hydrogen-bond acceptor and donor. This enhances the drug–polymer interaction, consequently resulting in an increased drug–polymer interaction. In the ASD+ polymer system, the addition of a third polymer can form hydrogen bonds with both the drug and the polymer. The binary system sometimes is unstable because of the lack of non-covalent bonding between the drug and polymer. Thus, adding the third polymer can form a hydrogen bond with the drug and polymer, leading to a more thermodynamically stable system. This system also resulted in a synergistic enhancement of amorphous stabilization and dissolution and precipitation inhibition and supersaturation [60,65]. Meanwhile, with the addition of a surfactant to the binary system, surfactant clusters would be adsorbed on the two polymer chains, which leads to increased viscosity and greater stability of the solid dispersion [121]. The ionic or hydrogen bonding between the water-soluble polymer and a third water-soluble excipient and the molecular interaction between the drug and polymer can stabilize the ternary solid dispersion system at varied pH levels [76]. The high viscosity and the interaction between the drug and polymer and a third component cause the mobility suppression of the amorphous drug, which contributes to the higher stability compared to the binary system. As a result, the TSD system exhibits enhanced physical stability, which is expected to surpass that of the binary system.

## 10. Conclusions

In conclusion, the recent literature contributes to elucidating mechanisms of TSD dissolution and drug distribution in an aqueous environment. TSDs emerged as appealing alternatives for surmounting the limitations associated with the utilization of binary systems, such as ASD and co-amorphous/cocrystal systems. The incorporation of a third component in binary systems has the potential to enhance their performance across various aspects, including solubility, wettability, dissolution profile, physical stability, and processability. However, there were challenges in the preparation of the TSD system, especially in choosing an appropriate third component and a preparation method. Moreover, due to TSDs being complex systems, it would be difficult to characterize the mechanism of the TSD system. Therefore, these could be challenges for researchers when developing an amorphous formulation of the TSD system. Furthermore, this study has contributed fundamental insights into the TSD system, which is significant when formulating strategies for enhancing the bioavailability of poorly water-soluble drugs.

## Figures and Tables

**Table 1:** TSD systems from ASD systems with added surfactants.

Drug	Polymer	Surfactant	Method of Preparation	Purpose	References
Manidipine	Copovidone	TPGS	Melt method	Improve Solubility	[36]
Curcumin	PVP	Tween 80	Rotary evaporation	Increase Dispersion, Solubility, and Stability	[37]
Valsartan	HPMC	SLS	Spray-drying	Increase Solubility (in vivo)	[38]
Docetaxel	Povidone K30	Sodium dodecyl sulfate	Spray-drying	Increase Solubility	[39]
Sirolimus	Eudragit^®^ E	TPGS	Spray-drying	Improve Solubility	[40]
Itraconazole	PVP VA64	Myrj 52	Rotary evaporation	Stability Evaluation	[41]
Itraconazole	HPMCAS	Poloxamer 188 and 407	Hot-melt extrusion	Improve Processability and Drug Release	[42,43]
Itraconazole	HPMC	Pluronic F68	Solvent evaporation	Improve Solubility	[44]
Gemfibrozil	PEG 6000	Sucrose laurate	Hot-melt extrusion	Improve Solubility	[45]
Paclitaxel	PVP K30	SLS	Freeze-drying	Improve Solubility	[46]
Itraconazole	PVP VA 64	Inutec SP1	Spray-drying	Improve Solubility	[47]
Itraconazole	HPMC	Poloxamer 407	Supercritical anti-solvent process	Improve Solubility	[48]

**Table 2:** TSD systems from ASD systems with added polymers.

Drug	Polymer	Polymer	Method of Preparation	Purpose	References
Tectorigenin	PVP	PEG4000	Rotary evaporation	Improve Solubility	[55]
Nilvadipine	Methylcellulose	Crospovidone	Rotary evaporation	Improve Solubility	[56]
Nifedipine	HPMC	Eudagrit^®^ S	Spray-drying	Improve Solubility	[57]
Cilostazol	HPMC	PVP	Spray-drying	Improve Solubility	[58]
Indomethacin	Eudragit E100	PVP K90	Rotary evaporation	Improve Solubility	[59,60]
Glyburide	HP-β-CD	Kollicoat^®^ IR/PVP	Freeze-drying	Improve Solubility	[61]
Ibuproxam	PVP	PEG	Co-evaporation	Improve Solubility	[62]
Griseofulvin	HPMC AS	PHPMA	Spray-drying	Improve Stability	[63]
Itraconazole	HPMC 2910 E5	PEG	Spray-drying	Improve Stability	[64]

**Table 3:** TSD systems from ASD systems with added excipients.

Drug	Polymer	Excipient	Method of Preparation	Purpose	References
Glycyrrhetinic acid	PVP	L-arginine	Hot-melt extrusion	Increase Solubility	[67]
Indomethacin	HPMC/Copovidone/Kollicoat^®^ IR	Hydrous silicon dioxide	Hot-melt extrusion	Improve Processability and Solubility	[68]
Telmisartan	PEG 6000	Magnesium oxide	Rotary evaporation	Increase Solubility	[69]
Tibolone	PVP	Silicon dioxide	Solvent evaporation	Sustained Release	[70]
Rebamipide	PVP VA 64	Sodium hydroxide (alkalizer)	Spray-drying	Increase Solubility	[71]
Troglitazone	PVP	Light anhydrous silicic acid	Co-milling method	Improve Stability	[72]
Celecoxib	PVP	Isomalt	Spray-drying	Improve Solubility	[73]
Cinnarizine	Soluplus^®^	Sorbitol/Citric acid monohydrate	Hot-melt extrusion	Improve Solubility and Stability	[74]
Rebamipide	Sodium alginate	Sodium carbonate	Spray-drying	Increase Solubility	[75]

**Table 4:** TSD systems from co-amorphous/cocrystal systems with added excipients.

Drug	Co-Former	Polymer/Excipient	Method	Result	References
Carbamazepine (CBM)	Citric acid (CA)	L-arginine (ARG)	Ball-milling	Improved dissolution profiles	[78]
Sulfamethoxazole (SMZ)	Trimethoprim (TMP)	Eudragit (EDE) and polyacrylic acid (PAA)	Melt-quenching	Improve the stability and area under curve (AUC)	[79]
Ketoconazole (KZN)	each oxalic (OXA), tartaric (TAR), citric (CIT) and succinic (SUC) acid	PVP	Spray-dried dispersions	Improved dissolution and physical stability	[80]
Indomethacin (IMC)	Saccharin	PVP	Spray-drying	Improved dissolution profiles	[81]

**Table 5:** A simple comparison of different kinds of ternary solid dispersion.

No	Component of Ternary Solid Dispersion	Advantages	Disadvantages
1	TSD systems from ASD systems with added surfactants	- The presence of a surfactant in the binary system can improve solubility and processability - Surfactants can inhibit thermodynamic and kinetic precipitation	- Nonionic surfactants could not be used in this system because they cannot form ionic bonds with the polymer chains - Reduces stability and dissolution by promoting the crystallization of the drug
2	TSD systems from ASD systems with added polymers	The addition of a third polymer enhances synergically amorphous stabilization and dissolution	The high viscosity of this system revealed the low wettability, leading to the slow dissolution rate
3	TSD systems from ASD systems with added excipients	The addition of a third water-soluble excipient can stabilize the system at varied pH levels	The large molecular weight and low water solubility of some excipients lead to the limitation of their application
4	TSD systems from co-amorphous/cocrystal systems with added excipients	The addition of a polymer can form complexes with the drugs, which enhances the stability and solubility of amorphous drugs	The addition of a polymer in the co-amorphous or cocrystal system can decrease the maximum achievable supersaturation, leading to a slow dissolution rate

**Table 6:** A brief summary of the characterization of ternary solid dispersion in the solid state.

Analytical Method	Purpose	Results	Conclusion	References
Fourier Transform Infrared (FTIR)	To analyze the intermolecular interactions between drugs and excipients in the TSD system	The shifting of the major absorption bands of drugs in the TSD system	Hydrogen-bond interactions between the drugs and excipients in the TSD system	[83,87]
Differential Scanning Calorimetry (DSC)	To detect the glass transition temperature (*T_g_*) of amorphous drug in the TSD system To analyze the intermolecular interactions between drugs and excipients in the TSD system	The presence of *T_g_* The difference in *T_g_* value between the amorphous drug and the TSD system	Amorphous drug in the TSD system Intermolecular interactions between drugs and excipients in the TSD system	[80]
Powder X-ray diffractometry (PXRD)	To analyze the amorphization of drugs in the TSD system	Halo patterns	Amorphous drugs in the TSD system	[93]
Thermogravimetric analysis (TGA)	To determine the composition of each component in the TSD system	The weight loss due to the degradation	Composition of each component in the TSD system	[97]
Solid-state NMR	To assess the molecular state of drugs in the TSD system	Broad peaks of drugs Downfield shift from amorphous drugs	Amorphous drug in the TSD system Intermolecular interactions between amorphous drugs and excipients in the TSD system	[105]
Dielectric spectroscopy	To measure the molecular mobility of drugs in the TSD system	α-relaxation time	Molecular mobility of drugs in the TSD system	[79,80]
Scanning Electron Microscope (SEM)	To examine the morphology of drugs in the TSD system	The particle size and shape	The particle size and shape of drugs in the TSD system	[80]

**Table 7:** A brief summary of the characterization of TSD systems after being dispersed in water.

Analytical Method	Purpose	Results	Conclusion	References
Dynamic Light Scattering (DLS)	To analyze the particle size distribution of drugs in the TSD system after being dispersed in water.	The size and PDI values.	Distribution of drugs in the TSD system after being dispersed in water.	[108]
TEM and Cryo-TEM Measurements	To determine the morphology of drugs in the TSD system after being dispersed in water.	The size and shape of nanoparticles.	The size and shape of drugs in the TSD system after being dispersed in water.	[108,109]
Atomic Force Microscopy (AFM) Measurements	To evaluate the topography and stiffness of the TSD system in an aqueous solution.	The size, shape, point of contact, and force of nanoparticles.	The size, shape, elasticity and stiffness of the TSD system in an aqueous solution.	[109]
Solution-State ^1^H NMR	To determine the hydrogen-bonding interactions between the drugs and excipients in the solution phase.	A shift of the peak to the shielded region in the NMR.	Hydrogen-bonding interactions between the drugs, and excipients in the solution phase.	[110]
Zeta Potential	To examine the dispersion stability of amorphous drugs in the TSD system after being dispersed in water.	The charge and surface zeta potential of the TSD system in the solution phase.	Good stability in the dispersed state.	[98]

## Data Availability

Not applicable.

## Abbreviations

The following abbreviations are used in this manuscript:

APIs	Active pharmaceutical ingredients
ASD	Amorphous solid dispersions
TSD	Ternary solid dispersions
NCE	New chemical entities
HPMC AS	Hydroxypropyl methylcellulose acetate succinate
PVP	Polyvinylpyrrolidone
PHPMA	Poly[2-hydroxypropyl methacrylate]
GF	Griseofulvin
HP-β-CD	Hydroxypropyl-βcyclodextrin
PEG	Polyethylene glycol
CBM	Carbamazepine
CA	Citric acid
ARG	L-arginine
SMZ	Sulfamethoxazole
TMP	Trimethoprim
EDE	Eudragit
PAA	Polyacrylic acid
AUC	Area under curve
KZN	Ketoconazole
OXA	Oxalic acid
TAR	Tartaric acid
CIT	Citric acid
SUC	Succinic acid
IMC	Indomethacin
FTIR	Fourier transform infrared
TEL	Telmisartan
DSC	Differential scanning calorimetry
*T_g_*	Glass transition temperatures
SIM	Simvastatin
Myrj 52	Polyoxyl 40 stearate
PM	Physical mixtures
PXRD	Powder X-ray diffractometry
BDQN	Bedaquiline fumarate
TPGS	Tocopheryl polyethylene glycol 1000 succinate
PLM	Polarized light microscopy
TGA	Thermogravimetric analysis
FEB	Febuxostat
EZ	Ezetimibe
ssNMR	Solid-state nuclear magnetic resonance
PBC	Probucol
HME	Hot-melt extrusion
P407	Poloxamer P407
τα	α-relaxation time
SEM	Scanning electron microscopy
DLS	Dynamic light scattering
SDS	Sodium dodecyl sulphate
MV	Mean volume diameter
PDI	Polydispersity index
BSD	Binary solid dispersion
cryo-TEM	Cryogenic transmission electron microscopy
MN	Mean number diameter
GM	Ground-mixture
AFM	Atomic force microscopy
SPD	Spray-dried sample
North 2	Indole2-carboxamide derivative
OM	Olmesartan medoxomil
MG	N-methyl-D-glucamine
MDP	Manidipine hydrochloride
ThSD	Thermal stress device
IDR	Intrinsic dissolution rate
SDs	Solid dispersions
ITZ	Itraconazole
HPC-SSL	Hydroxypropylcellulose
